# “I’m going to change the WIFI password if you don't go outside!”: a qualitative examination of changes in parenting practices over the course of the COVID-19 pandemic

**DOI:** 10.3389/fspor.2023.1270516

**Published:** 2023-12-12

**Authors:** Derek Paterson, Mark R. Beauchamp, Louise C. Masse, Sarah A. Moore, Guy Faulkner

**Affiliations:** ^1^ School of Kinesiology, The University of British Columbia, Vancouver, BC, Canada; ^2^ School of Population Health, University of British Columbia, Vancouver, BC, Canada; ^3^ BC Children’s Hospital Research Institute, Vancouver, BC, Canada; ^4^ School of Health and Human Performance, Dalhousie University, Halifax, NS, Canada; ^5^ Department of Pediatrics, Dalhousie University, Halifax, NS, Canada

**Keywords:** COVID-19, children, parenting practices, physical activity, screen time

## Abstract

**Introduction:**

Parenting practices are an important influence on the movement behaviours of children. Parenting practices are shaped by various contextual factors (e.g., culture, sociodemographic, community) and are susceptible to external pressures, such as health crises like the COVID-19 pandemic. Situated within the revised Family Ecological Model, the purpose of this study was to qualitatively explore changes in parenting practices over time in relation to the evolving nature of this global event.

**Methods:**

One-on-one semi-structured interviews with 40 parents of children (aged 7–11) in three Canadian provinces were conducted in August, 2021. A narrative thematic analysis was conducted to develop themes mapping changes in parenting practices and organize the temporal patterns of these changes into shared case trajectories over time.

**Results:**

Four themes were constructed to highlight changes in parenting practices: (1) Screen time permissiveness, (2) Force and coercion, (3) Agents of unstructured physical activity, and (4) Stepping back from structured physical activity. These themes were organized into three distinct case trajectories that each represent a shared, chronological narrative for how the first 18 months of the pandemic were broadly experienced by parents. The three trajectories were characterized by: (1) Resilience (2) Enduring impact and (3) Adaptive growth.

**Discussion:**

Parenting practices were changed in response to circumstances in both temporary and enduring manners that may continue beyond the pandemic. Further research is needed to longitudinally assess these trajectories in order to support families and enhance understanding of parenting practices in challenging circumstances.

## Introduction

There is considerable evidence demonstrating associations between movement behaviours (i.e., physical activity, sedentary behaviour, sleep) and the health of children (1). A complex interplay of individual, social, environmental, and political factors have been found to be influential in the movement behaviours of children (2). A rapidly accumulating body of evidence has pointed to the strong influence of parenting and family factors on children's behaviours, particularly as it relates to physical activity (3, 4). The influence of parenting practices, goal-directed behaviours used to influence children's behaviours (5), have become a key element of inquiry into the determinants of children's movement behaviours. Evidence consistently demonstrates that the specific practices deployed by parents, such as role modelling, encouragement and logistical support, are instrumental in the movement behaviours of their children (6).

According to the revised Family Ecological Model (RFEM) (3), parenting practices can be understood as existing within socio-ecological contexts that are composed of influences from community, organizational, media, policy and family-historical factors. These factors may in turn interact to mould the specific behaviours and practices of parents and ultimately those of their children (3). For example, a parent's work demands (ecological context) may influence their self-efficacy in restricting screen time which in turn may shape their practices to create physically active alternatives for their child. With consideration for the logic model proposed by the RFEM, changing circumstances across any layers of the social-ecological context could be a catalyst for collateral changes in parenting practices and ultimately, children's movement behaviours.

During March of 2020, the outbreak of the COVID-19 virus was declared a global pandemic (7), disrupting the lives of children and families worldwide. Since the declaration of the pandemic, increasing evidence has demonstrated significant declines globally in physical activity and rises in sedentary behaviour/screen time amongst school-aged children specifically during the first six months of the pandemic (8). A nationally representative survey of Canadian families reported less than one quarter (23.8%) of school-aged children (aged 5–11) were adhering to 24-h movement guidelines for moderate-to-vigorous physical activity (MVPA) and just 16.5% for screen time during the initial wave of the pandemic in April of 2020 (9). Six months later, in October of 2020, there was even lower adherence to 24-h MVPA guidelines (17.5%) while the proportion meeting recreational screen time guidelines rose to 35.4% (10). These results highlight the immediate and temporally evolving impact of the pandemic on the movement behaviours of children and youth living in Canada.

A scoping review demonstrates the indirect, potentially long-term health consequences of prolonged COVID-19 restrictions on children by demonstrating extensive adverse changes in movement behaviours (8). However, this evidence has been largely underpinned by descriptive, atheoretical research to this point and the data are often presented without description of the socio-ecological contexts that participants are living. Through the lens of the RFEM, it would be expected that the experiences of families during the pandemic have varied based on their individual life circumstances. There have been variable effects on movement behaviours of child populations based on regional restrictions (11) and environmental factors (12) while some children even increased or maintained their physical activity (13). With restrictions to a variety of spaces and reduced opportunities for physical activity, and government and health officials urging or mandating shifts to stay-at-home routines, it was expected that in many contexts, parents have faced circumstances that facilitate inevitable (mal)adaptation and change in their practices and parenting strategies. Limited research on parents' experiences during the pandemic has highlighted the difficulty parents faced early in the pandemic in being able to support their children's healthy movement behaviours (14) although there has been little explicit focus on changes in parenting practices *per se* in existing qualitative literature (15). As the pandemic progressed into its second year, with the nature of restrictions fluctuating over that time, there was a pressing need to consider how families adapted over this time course. Accordingly, through qualitative semi-structured interviews with parents (of children aged 7–11), the purpose of this study was to explore the impact of the pandemic on movement behaviour parenting practices and examine the extent to which practices change in response to temporally changing circumstances.

## Methods

This study adopted an interpretive paradigm through a worldview in which the researcher assumes that the “real” world cannot be fully accessed and knowledge about it is socially influenced and subjective. The idea of a single reality is rejected and knowledge is assumed to be context-dependent (16) meaning that findings will vary based on the contexts where the data are situated. While no one truth can ever be arrived at, there is the notion that certain knowledge may be true in particular contexts (16).

### Participants and recruitment

This study deployed a non-probabilistic purposive sampling technique from within a larger national sample of Canadian families of school-aged children who participated in a quantitative survey that assessed changes in children's movement and play behaviours six months into the pandemic (10). To be included in the study participants were required to be an English-speaking parent of at least one child aged 7–11, and located in either British Columbia, Ontario, or Nova Scotia. We set an initial target to conduct 12–15 interviews with participants located in each of the three included provinces for an initial pragmatic target of 36–45 interviews. This target was set for, and constrained by, pragmatic limitations rather than as an expected point of saturation; a concept not appropriate for determining sample size within qualitative, constructivist paradigms (17). A final sample of 40 parents were reached at the point when researchers felt the data collected were sufficient to support analyses. Determining how many interviews to conduct was an ongoing and reflexive process that required constant consideration of the richness of the data being collected and the evolution of our interpretations of the data. A sample size of 40 can be considered a relatively large sample particularly in comparison with other qualitative studies with parents regarding movement behaviours during COVID-19 (*n* = 21 (18); *n* = 29, Riazi et al., 2021; *n* = 20 (19). The final sample included 27 identifying as mothers. These parents were evenly distributed across 3 provinces in Canada: British Columbia (13), Ontario (13) and Nova Scotia (14). Of the 40 children of focus, 15 were identified by parents as girls, the other 25 as boys. A complete overview of participant demographics can be viewed in Supplementary File S1.

### Data collection

The study was approved by the Institutional Behavioural Research Ethics Board (#H20-01554). Data for this study were collected through one-on-one semi-structured interviews with parents in August of 2021. Semi-structured interviews were a suitable method of data collection, given the qualitative paradigm of the study. Semi-structured interviews facilitate participants in providing rich descriptions of their lived experiences and contexts and allow the participant and researcher to co-construct meaning through their interactions. The flexibility of semi-structured interviews was an asset given the novelty of the pandemic and therefore the potential for new, unplanned avenues of meaning to be explored. After providing consent, interviews were conducted and recorded via the Zoom platform. Interviews lasted on average 1:00:44 and were transcribed manually and verbatim. The interview guide can be viewed in Supplementary File S2.

### Analysis

A thematic narrative analysis (20) was conducted; a combined technique, incorporating a reflexive thematic analysis (21) and narrative analysis (22). The hybrid use of these methods has been used successfully in previous qualitative research (20, 23) and has been endorsed as compatible methods by advocates of reflexive thematic analysis, Braun and Clarke (17). Reflexive thematic analysis enabled the identification of themes related to changes in parenting practices over the first 18 months of the pandemic and explored similarities and differences across the sample. Coding was performed by the first author both deductively and inductively and was done using hardcopies and the use of NVivo software. The incorporation of a narrative analysis was used to examine temporal patterns, by facilitating chronological organization of participant accounts (24) and the themes within them, previously identified by the thematic analysis. This approach to narrative analysis followed a thematic model (22) focusing on the content of the narratives more than how stories were told (24). Here, a “restorying” process was carried out in order to sequence the thematic narratives told by participants into chronological “stages” of their accounts of living through the pandemic. The first stage in doing this was another round of coding with a focus on temporal interpretations of the transcripts (24). These codes were then collected into folders in Nvivo and used to write a narrative summary of each participant's temporal trajectory regarding changes in parenting practices. Then, individual trajectories were sorted into groups based on similarities in the temporal order of events related to the themes. Within each of these groupings, both the summaries and the codes/data were referenced to write a single, “meta” trajectory summary (20) of a fictional, case family that captured the amalgamated, temporal experiences of these parents over the first 18 months of the pandemic. To enhance trustworthiness, throughout data collection, analysis and writing of the manuscript, co-authors acted as “critical reviewers” (25) to encourage further reflection and alternative interpretations of the data.

## Results

Following the presentation of four overarching themes, each of the case trajectories are presented below.

### Theme 1: screen time permissiveness

Most parents described being more permissive towards their children's screen-based behaviours by relaxing their use of rules and restrictions. The extent of permissiveness ranged from a “loosening of the reigns” as with NS14, “*Yes we gave him a little more screen time,”* to abandoning any limitations “*Oh, no, yeah those rules went right out the window”* (NS5). Several contextual processes were reported to drive this change. Prior to lockdowns, the busyness of daily life acted as an organic regulator of children's screen time: “*Their screen time is up because other things are down” (NS4).* BC12 similarly noted how they had not previously needed to make conscious efforts to limit screen time: “*We weren't really, really strict on it, but he was so engaged with everything else that we didn't really have to monitor it.”* For many parents, this bred inexperience taking direct actions to limit screen usage through other behaviour substitutions: “*The day was so long and … for me to be able to entertain them for such a long period of time was really, really hard.”*

Permitting screen time was also recognized as an effective “babysitting tool”, allowing parents to attend to other responsibilities including their jobs: “*A typical day was me working and him downstairs in his little mancave watching YouTube and playing Xbox…I just gave it to him so I could keep him out of my hair so I could work” (BC4).* Finally, there was a palpable empathy that parents displayed for the child experience of lockdown:

*I knew the toll COVID was taking psychologically as well, I was like I'm not going to be a big disciplinarian here. It is end times, does it matter if he is on his Switch for 2 h that day? No (ON7)*.

This sense of empathy justified screen time permissiveness in acknowledging the challenges of the pandemic for children and their families.

### Theme 2: parent initiation: forceful demands and coercion

Being inside on a device had become a newly developed comfort zone for many children, lingering beyond the removal of restrictions. A common concern raised by parents was a belief that their children would never do anything other than play on their screens if not for parental intervention: “*To be honest with you, if left to their own devices, they would literally just sit on a screen all day” (ON14).* This appraisal of their child's excessive screen usage and lack of initiative led some parents to resort to coercive parenting practices to initiate physical activity as a perceived counteractive measure:

*I've been more strict just because I've allowed them more leniency in their screen time so I feel like this is my counterbalance. Like if you are going to have more screen time then I am going to be more insistent that we do get out and do something (ON8)*.

In some cases, this meant that parents felt they needed to use insistent and demanding language, forcing outdoor time by framing it as a requirement rather than a suggestion. NS1 exemplifies how this was perceived to be the only option for them: “*It came to the point he wasn't going outside unless he was told … we would say we are going now, you must come with us. It has been a struggle because of the electronics.”* However, for many other parents such as NS9, screen time was leveraged as a tool for reward/punishment to coerce outdoor time and physical activity: “*It was like well if you don't go and play, you know you aren't going to have game time on your PS4 and you won't talk to your friends.”* This was typically perceived as an effective strategy for influencing physical activity and some parents were willing to take “extreme” measures when needed like BC12's approach: “*Look, I'm going to change the password on the WiFi if you don't go outside.'*

### Theme 3: agents of unstructured physical activity

Despite most parents dealing with new challenges related to screens, it was also common for parents to adopt new or enhanced parenting practices related to their own active involvement and support of their child's unstructured physical activity. Career disruption left room for some parents to seize the opportunity to reinvest time and energy into the health and leisure of their family:

*The overall shift is that we are somewhat less career focused and a little more family focused and have invested more time than we might have otherwise into his activities…We've had more time to become more active ourselves again, and so one of the things is just leading by example (BC1)*.

Here, BC1 exemplifies how some parents engaged in new parenting practices such as role modelling and co-participating in unstructured physical activities. Others like NS12 replaced the roles of others: “*I was more of a mom sitting on the sidelines cheering her on, now I am the coach and the cheerleader.”* Looking for novelty paved the way for new family activities such as with ON1: “*Roller blading has actually had a resurgence,*” or ON6: “*Kayaking would be one thing that we did pick up during the pandemic*.”

Many parents generated novel opportunities for physical activity by purchasing equipment to support unstructured play. This may have been as minor as ON3 purchasing some new loose parts, “*We bought some ropes so they can jump rope. Trying to find a way to do something.”* or as extensive as NS4 completely renovating their yard to support play: *“We put a concrete slab in the backyard and made a basketball court for them and this year we actually put in a pool.”*

### Theme 4: stepping back from structured physical activity

Despite availability following lockdowns, many families opted not to return their children to the structured activities they had previously participated in. Instead, it was common for families to delay returns or to have reduced the emphasis on structured physical activities. For some, this was purely a case of lingering barriers produced by the pandemic such as ongoing COVID-19 fears or frustration with the logistics of navigating “opening up”:

*It's become a real bureaucratic nightmare of clearances and oh god, everything now involves a call just before you start to make sure everybody*’*s not sick and you show up and God forbid … “Do I really want to go through this? …It*’*s not worth it (BC14)*.

For many other parents, reductions in structured activity enrolment were a product of intrinsic shifts in lifestyle preferences. The pandemic for some parents had provoked self-reflection regarding the frenetic nature of their lives and a chance to appreciate a slower pace of life. As NS6 reflected:

*It really did slow things down a lot and put into perspective the rat race of life you know? Pre pandemic, you are running here and there and they are in this and that…Everybody had to take a breather you know?*

NS3 echoed this desire to maintain a slower, family-centred lifestyle moving forward:

*We were living such a hectic life…Now it is a slower pace lifestyle and I think we are all happier because of it. I am hoping going forward that we can maintain that rather than going back to just busy every day, all day before the pandemic. Just enjoying what is in our own yard*.

### Meta trajectories

The following section outlines three distinct trajectories detailing the commonly shared temporal patterns of change in parenting practices (see Supplementary File S1). These trajectories were constructed as meta representations of similar parental experiences. The trajectories highlight distinct temporal patterns in how changes to parenting practices occurred over the course of the COVID-19 pandemic.

### Trajectory 1: “back to business as usual”

The first trajectory depicts a family whose background and living circumstances fostered conditions for greater resilience throughout the pandemic. The parents expressed strong beliefs in leading an active and outdoor lifestyle for themselves and their family: “*I grew up as a child outside from the time I woke up to when I went to bed. That is the lifestyle I wanted for my children and I incorporated that in their lifestyle” (NS11).* As an advocate of the outdoors, the parent influenced their children's movement behaviours prior to the pandemic in different ways but with emphasis on supporting informal outdoor activities through co-participation, modelling, and autonomy promotion. These parenting practices were enabled through both possession of outdoor space on their property (e.g., backyard) and living near greenspaces providing opportunities for active leisure. A parent reflected on these privileges: “*We have a garden. Gardening, helping out…we live near a lake, so we’re blessed with that ability to swim, go for a boat ride or a canoe. That is normal for us” (NS10)*.

With the initial restrictions, this family lost access to structured physical activities and facilities like all trajectories. However, the access to, and experience with, informal outdoor activities helped mitigate the magnitude of the disturbance*. “It was the same activity wise because we were getting outside in our yard” (NS11).* This is not to say they were unaffected: “*Obviously we were restricted to some degree but we just made it work” (NS15).* While many sources of physical activity were lost, the parents were able to maintain some domains of support through their familiarity with facilitating family physical activities. “*They haven't been less active. Nothing has really changed. I mean we continue to do our thing and come up with ideas. I’m an adventurist, I’m always coming up with a plan each day and they just love that' (NS15).* With parks and other outdoor spaces mostly closed during the initial lockdown, these parents' practices were most heavily curtailed early on. During this initial wave of the pandemic, the closure of schools created conflicts between employment and parenting demands leading to new screen time permissiveness. As one parent explained “*I mean I do have a full-time job, I’m on the phone quite a bit so I need to be behind closed doors. It is what it is right?’ (NS15)* referencing the inevitable nature of some early screen time allowances.

As time passed, circumstances trended back to “normal’. Restrictions lifted, and even in later lockdowns, the closures to activities and outdoor spaces were less severe. In addition, the parents held a low concern for the virus: “*I’m a little blunt I guess, just suck it up and let's go do this, don't worry about public spaces, we're going to be safe. I do like to bring them out and say, “Hey the world is still moving”' (BC5).* This enabled a quicker return to public and group activities than for other more cautious families: “*The basketball program they’re in…whenever it got re-opened we registered them” (ON10).* Along with the return of typical activities and schooldays, came a restoration of previous strictness and control over screen time: “*When school went back in I said the rules were reinstated” (NS5).* By this point, in the autumn of 2020, the practices of the parent to support their children's movement behaviours had stabilized with one parent declaring that “*It's back to business as usual” (NS5)*.

In the case of this family, resilience was exhibited with parenting practices elastically returning to an initial state after physical distancing mandates were eased. The parents had pre-established parenting practices that were adaptive to the circumstances of the pandemic such as co-participation, modelling and encouraging outdoor play. For this reason, parents did not have to make radical adaptations to support their children, and any adaptations that did occur were temporary.

### Trajectory 2: “stuck in a rut”

The second case family experienced a trajectory that was characterized by enduring modifications in parenting practices. Despite the abruptness and strictness of the first lockdown, the lockdown brought an element of novelty early on enabling families to occupy time by co-participating in activities together, such as walks and bike rides or informal play in and around the home. “*We put our trampoline up early even though it was cold, so they were all out playing and doing that stuff and to them it was all exciting and different because they are so used to school and school nights’ (ON12).* However, the limited options for active and outdoor time could only sustain the entertainment of the child for a finite period of time. “*I got some bocce balls and then we would spend the afternoon tending to the garden and playing games in the backyard and he was fine for about a month” (BC12).* After approximately a month, the interest and desire of the child to engage in active play dwindled as available activities became repetitive and dull.

*As it dragged on it got a little more monotonous because we were still at home and there was the same thing every day so days dragged into weeks and even with the pool outside they got bored very easily because we couldn't do anything else (ON12)*.

At this point, the parent became more permissive towards screen time as the child's preferences became centred around devices: “*It's boring for a kid right? There's only so many bocce ball or badminton games that a kid can play. That's not exciting, that's not Halo, that's not GTA, that's not what they want to do” (BC12).* With jobs to attend to, and empathy for their children, screen time permissiveness became established in their parenting style, with the child given free-reign over regulating their own screen usage. “*I didn't give any kind of restrictions on time that they can play…I just let them play what they wanted” (BC3).* Even as lockdown restrictions were loosened in the first summer and autumn, and activities became available, permissiveness remained elevated: “*The screen usage will stay, realistically, kind of high for a while because it is habits we have formed” (ON1)*.

With children developing the habit of in staying indoors, it became difficult for the parent to remotivate their child to engage in activities outside the house and take initiative to do so. “*Oh hell no it's not the parents. We want these kids outside, it's just that they’re stuck in a funky rut where it's just easier to be at home” (BC12).* Into the latter stages of 2020 and into the spring of 2021, the parent grew dissatisfied with the lingering preferences of the child to stay home and began to resort to forceful and insistent practices to initiate outdoor time: “*It was definitely something that we had to push, and we have to continue to push because he's so used to being in the house doing what he wanted to do” (NS1).* As the parent noted here, this use of forceful parenting remained in practice at the time of interview, with potential to continue indefinitely.

Exacerbating the narrative for this family was the lingering perception of barriers prohibiting re-entry into structured physical activities. After 18 months, programs and leagues were operational, but ongoing concerns with COVID-19 risks and distrust of service providers fostered an unwillingness to re-enrol the child.

*Yeah, I mean normally he would be in a day-care at the school and that keeps him active but currently he is home and, on the screens so much because what can you do right? I don't trust the pandemic right now. Some people are putting their kids in day camps and what not, I don't feel safe (ON7)*.

As a result, screen time permissiveness persisted over time in parallel with new forceful and demanding parenting practices deemed necessary to influence child physical activity. For this trajectory, screen time permissiveness and forceful demands have been maintained long after the initial implementation of restrictions.

### Trajectory 3: “upside to slowing down”

Prior to the pandemic, the parents of this family unit were heavily occupied with the workloads of their job and careers. They navigated parenting alongside this career focus via regular reliance on structured programs and after school activities: “*I think we figured out one day that six days a week they were in organized physical activity, outside of school” (BC14).* With the bulk of their lives spent engaged in routines outside of the home, the initial onset of restrictions in the first wave felt particularly disruptive and abrupt for this family*. “That is probably where we felt the most constricted because everything we were so used to doing stopped all at once” (BC4).* With participation in structured activities so embedded within routines, this fostered a lack of preparation initially for daily life restricted to the home. “*I think the initial week or so was pretty much do whatever you want, we’ll figure this out but yeah they went from a lot of activity to just about zero initially” (BC14).* The early “do whatever you want” mentality was embodied by this parent through elevated screen time permissiveness as well as reduced efforts to facilitate physical activity brought on by closures and COVID concerns.

However, transitioning into the summer, a pair of processes began to inspire change. First, with case counts declining perceptions of risk were changing. “Early May was when we stopped giving a s**t. I was done. At that point we started going to the park and seeing family again and that made things easier” (ON6). In addition, parents were also beginning to come to terms with the long-term nature of the pandemic and the need to proactively find ways to handle it. One parent discussed this change in mentality: “*I think it was as we began to realize just how long the pandemic was going to last, and that we were going to have to just adapt to our new lifestyle that we started to pick up some new activities” (BC1).* As these changes occurred, the parents stepped into new roles to influence the physical activity of their children, something that was made possible by the career disruption faced by the parents.

*They cut back our hours, like I went down to minimum part-time hours. I would basically work a couple days a week… me having a little more time off and the weather has been great so it's been pretty easy to get out and do stuff (BC11)*.

With job demands reduced, and the financial stability to support it, the parents were able to reinvest time into their family health and leisure, embracing family physical activity and becoming an active leader for their child through co-participation, role modelling and teaching: “*We would get him out of the house whether it was a bike ride or out on the street playing some sport, but there was a lot more time to teach various things” (BC11).* Living in a detached house in a safe, spacious neighbourhood helped support participating in these unstructured activities. This new time spent embracing being active with their children was enjoyed by the parents and led to restructuring of priorities in their life.

Physical activity had become more central to family time together, and the parents were relishing a more informal, family-centred lifestyle. As a result, the family did not rush out to re-enrol the child in the same breadth of structured activities in which they had participated previously. Reflecting on the value of this time, the parent was disinterested in falling back into the role of daily chauffeur.

*We are kind of going to take things a little bit slower…I find when there was too many organized sports it was just too much for me because I don't want to have to come home and drive them somewhere every day and it was just too much stress for them (BC6)*.

There was a clear sense that the entire family unit was happier with a more balanced lifestyle: “*I think one of the things we took away from this whole experience was how important it is to balance life and career…. There has been an upside to slowing down” (BC1).* With this family, there was a sense of positive growth in physical activity parenting practices. The parents became more actively involved in supporting the physical activity of their children and there was a desire to continue to do so indefinitely, displacing the facilitation of daily structured activities that dominated beforehand. In sum, this trajectory reflected the evolution of a growth-oriented approach to parenting that prioritized work-life balance and encouraging novel ways of supporting child PA.

## Discussion

The implementation of public health measures in response to the COVID-19 pandemic presented an opportunity to examine how parenting practices adapt over time. In line with the RFEM (3), changes to family ecology including parent job characteristics, reduced accessibility of community programs/services and safe play areas, all contributed to modifying parental strategies to influence child physical activity and screen time. These modifications were characterized by four themes: screen time permissiveness, force and coercion, active agents in unstructured activities, and stepping back from structured physical activity. Three trajectories were constructed as meta-representations of similar parental experiences to highlight distinct temporal patterns in how changes to parenting practices occurred over time.

Trajectories two and three both represent parents whose practices reshaped in potentially enduring manners. In most cases, parenting practices exhibited some degree of plasticity, retaining elements of their new state even after public health restrictions had been eased. The present study makes novel contributions by highlighting the changeability of parenting practices beyond those that might be expected to developmentally adjust as their child ages. To date, very little research has examined the evolving nature of parenting practices over time. A single longitudinal study examined parenting practice relationships with child physical activity and screen behaviours and found that child movement behaviours at age 5 predicted parenting practices at age seven (26). Further research is needed to understand broader temporal trajectories of parenting practices and the factors that may predict them in a non-pandemic context.

One concerning pattern found in the temporal trajectories constructed in the present study was the widespread development of screen time permissiveness. Permissiveness towards screen time early in the pandemic was consistent with existing qualitative literature (27). The present study advances our understanding of pandemic impacts by illustrating the enduring nature of these changes. For many families (trajectories two and three), screen time permissiveness had become consolidated beyond the relaxing of restrictions as parents often felt that the habits had formed and screens had become integrated within their daily lives beyond the point of no return. With ongoing permissiveness, screen time is likely to remain elevated among most children and it is possible that the pandemic has spurred a new standard for daily screen time amongst this cohort of school-aged children (10). Given that this study's data were also collected in the summer of 2021, it is important for research to continue to track screen usage over time to assess its ongoing trajectory. The trajectories suggest that screen time is likely the most important target for future intervention work involving young children. Interventions may need to target the family unit and focus on effective negotiations for limiting screen time and balancing it with physical activity.

Of particular concern are the parents (trajectory two) who reacted to the normalization of screens in their family by increasing their use of demanding and coercive physical activity parenting practices. Child preferences are reflected in the ecology component of the RFEM and may represent an additional avenue through which the pandemic has impacted parenting practices. Similar to Szpunar and colleagues who reported that parents saw screen time as a barrier to physical activity during the pandemic (28), parents in this study attributed forceful/coercive practices to the perception that their children now default to screen usage and no longer self-initiate physical activity. However, the establishment of forceful and coercive parenting over time as a response is a novel finding in the pandemic literature on parenting. The use of demanding/controlling practices has been related to lower child enjoyment and levels of physical activity (29). Furthermore, using screen time to control behaviours or as a reward has been linked to greater screen time (30). Given these concerns, longitudinal research is needed to further understand the factors that may influence the development of forceful and coercive parenting practices.

Theme 2 (force and coercion) highlights an interplay between physical activity and screen time parenting practices. Not only had dissatisfaction with screen time allowances prompted increased efforts to control physical activity by parents, but screen time permission and restrictions were repeatedly used to coerce children into physical activity participation. To this point, little research has examined the relationships between the parenting practices of these two movement behaviours. Neshteruk et al. (31) showed that parents who were permissive towards screen time also scored low on practices to support physical activity. However, this analysis did not consider forceful and coercive parenting practices and did not address the interactions or directionality of different practices. Further research is needed to understand the ways in which specific physical activity and screen time parenting practices may influence one another and how their use in combination may influence children's movement behaviours. How parents negotiate the interaction between child screen and physical activity behaviours may itself be a distinct parenting practice worthy of further investigation. Enhanced understanding of relationships between child physical activity and screen time could inform the development of intervention work that focuses on balancing both behaviours rather than attempts to change one or the other in isolation.

It is noteworthy that many parents reported positive growth in their parenting practices. It was a common theme for parents to have increased their participation in unstructured physical activity as a family, in line with other qualitative literature (e.g., 18). Within this theme, the growth of physical activity parenting practices such as non-directive support (co-participation, role modelling) and autonomy support (teaching, encouragement) were evident. The present study contributes to the literature by presenting evidence that the growth in these domains of parenting practices has exhibited plasticity in some cases (trajectory three) and may be sustainable over time after the pandemic. However, it is essential to acknowledge how contextual factors have created unequal opportunities for families. Parents represented in trajectory three were supported by job characteristics (e.g., reduced hours, flexibility, work from home etc.), access to play areas and resources, and the lack of competing priorities (economic stress, covid-concern) to be able to invest in an active family lifestyle. In contrast, trajectory two parents may have been limited in their capacity to adapt by one or more of these factors. While it is important to promote and support families to be active together and participate in outdoor play at home (32), future research, policy, and practice must consider how to be equitable and inclusive of families with the least access to do so. It was evident that safe play areas were valuable to families who could access them during lockdowns. It is important in the event of future lockdowns that messaging and mandates do not discourage or prevent the safe use of outdoor spaces. Additionally, the results reinforce the importance of equitable urban planning that encourages outdoor activity and does not exclude neighbourhoods, particularly in low-income areas, from proximity to amenities such as parks and trails.

The emergence of unstructured family activities was often closely related to the reduced use of structured programs and sports reported by trajectory 3 families. Elliot and colleagues' study of youth sport in Australia (33) reported comparable patterns in youth athletes not returning to sports in the wake of the pandemic. Similar to the present study, Elliot and colleagues referenced development of family connectedness along with other lingering logistical barriers related to the pandemic (33) as factors influencing this trend. Further research is needed to monitor ongoing youth sport participation trends. While many parents reported replacing structured activities with family activities, device-measured physical activity data may be needed to determine the extent to which this substitution affects time spent engaged in physical activity.

### Strengths and limitations

There are several limitations of the research that should be acknowledged. First, with interviews taking place in the summer of 2021, participants were asked to recall the timeline of events occurring over the previous 18 months. This may have limited the ability to provide rich accounts of specific events. It is also likely that developmental changes of the child over those 18 months played a role in the changing attitudes and behaviours of parents, particularly towards regulation of screen time. Second, the conduct of interviews over a virtual platform presented limitations on the quality of data collection. Without in-person interaction, many elements of effective communication and rapport-building were constrained.

These limitations should be considered in light of the strengths of the study. While much research has been generated regarding the impact of COVID-19 on the movement behaviours of children (8), to our knowledge this is the first study to focus on changes in physical activity and screen time parenting practices during this global event. One strength of this study was the diversity captured in the sample. This included an even distribution of participants from three different provinces with a balanced representation of child gender and age, parent gender, family size and household income. Another strength was in the thematic narrative analytic approach which involved both reflexive thematic analysis (21) and narrative analysis (22). This enables us to shed unique light on how parenting practices changed among families during the first 18 months of the COVID-19 pandemic.

## Conclusion

In summary, this study demonstrates that parenting practices changed throughout the COVID-19 pandemic. Parenting practices are susceptible to influence from external factors and can be modified in both the short and long term and those changes can be characterised through positive or negative trajectories. Future research is necessary to assess the ongoing temporal trajectories of parenting practices and their impact on children's movement behaviours in the aftermath of the pandemic. This will help develop strategies to support families now and in preparation for future public health crises.

## Data Availability

The dataset presented in this article is not readily available because of concerns about participant privacy and confidentiality. Requests to access the dataset should be directed to the corresponding author.
