# Supplementary material for: “I’m going to change the WIFI password if you don't go outside!”: a qualitative examination of changes in parenting practices over the course of the COVID-19 pandemic
Source: Front Sports Act Living. 2023 Dec 12;5:1270516. doi: 10.3389/fspor.2023.1270516 (PMC10773578; doi:10.3389/fspor.2023.1270516)
Supplement: Supplementary file 1 [file Table1.docx]

Supplementary File 1: Participants

| Case | Trajectory | Child  Age | Child Gender | Age  /Sibling | Marital status | Income | COVID-19 Concern | | Dwelling type | | Parent | |  |
| --- | --- | --- | --- | --- | --- | --- | --- | --- | --- | --- | --- | --- | --- |
| BC1 | 3 | 7 | B | None | Married | $150,000 - $250,000 | Somewhat concerned | | Detached house | | Father | |  |
| BC2 | 1 | 10 | B | None | Married | $50,000 - $75,000 | Very Concerned | | Low rise Apartment/Condo | | Father | |  |
| BC3 | 2 | 11 | B | None | Single, never married | $25,000 - $35,000 | Somewhat concerned | | Something else | | Mother | |  |
| BC4 | 3 | 9 | B | 5B | Married | $150,000 - $250,000 | Somewhat concerned | | Detached house | | Mother | |  |
| BC5 | 1 | 10 | G | 6B | Separated | $125,000 $150,000 | Not concerned | | High rise Apartment/Condo | | Mother | |  |
| BC6 | 3 | 10 | B | 8B | Common law | $50,000 to $75,000 | Very Concerned | | Townhouse | | Mother | |  |
| BC8 | 2 | 10 | G | 8G | Married | $75,000 $100,000 | Very Concerned | | Semi-detached house | | Father | |  |
| BC9 | 3 | 7 | G | 5G, 2B | Married | $25,000 $35,000 | Very Concerned | | Low rise Apartment/Condo | | Mother | |  |
| BC10 | 2 | 10 | G | None | Married | $150,000 $250,000 | Very Concerned | | Detached house | | Mother | |  |
| BC11 | 3 | 8 | B | 18G, 20G | Married | $100,000 $125,000 | Not concerned | | Detached house | | Father | |  |
| BC12 | 2 | 11 | B | None | Married | $125,000 $150,000 | Very Concerned | | Detached house | | Mother | |  |
| BC13 | 1 | 9 | B | 6G | Married | $125,000 $150,000 | Somewhat concerned | | Detached house | | Father | |  |
| BC14 | 3 | 11 | B | 9B | Married | $125,000 $150,000 | Very Concerned | | Townhouse | | Father | |  |
| ON1 | 3 | 11 | B | 7B, 9B | Married | $35,000 $50,000 | Somewhat concerned | | Detached house | | Mother | |  |
| ON2 | 2 | 7 | B | None | Common law | $35,000 $50,000 | Somewhat concerned | | Semi-detached house | | Mother | |  |
| ON3 | 1 | 11 | B | 6B | Married | < $25,000 | Not concerned | | Semi-detached house | | Mother | |  |
| ON4 | 3 | 7 | G | None | Married | $50,000 - $75,000 | Very Concerned | | Detached house | | Father | |  |
| ON6 | 3 | 10 | B | 6G | Common law | $25,000- $35,000 | Not concerned | | Semi-detached house | | Mother | |  |
| ON7 | 2 | 8 | B | None | Divorced | $50,000- $75,000 | Very Concerned | | High rise Apartment/Condo | | Mother | |  |
| ON8 | 3 | 7 | B | 5G, 3G, 1G | Married | $100,000- $125,000 | Somewhat concerned | | Detached house | | Mother | |  |
| ON9 | 3 | 10 | G | 8 weeks | Engaged | $100,000 - $125,000 | Somewhat concerned | | Detached house | | Mother | |  |
| ON10 | 1 | 8 | B | 12B | Married | $75,000 - $100,000 | Somewhat concerned | | Detached house | | Father | |  |
| ON12 | 2 | 10 | G | 6G | Common law | $50,000 - $75,000 | Very Concerned | | Detached house | | Mother | |  |
| ON13 | 2 | 12 | B | 6B | Married | $75,000 - $100,000 | Very Concerned | | Semi-detached house | | Father | |  |
| ON14 | 3 | 10 | G | 8G | Divorced | $75,000 - $100,000 | Somewhat concerned | | Detached house | | Father | |  |
| ON15 | 2 | 8 | G | 6G | Married | $150,000- $250,000 | Very Concerned | | Detached house | | Mother | |  |
| NS1 | 2 | 11 | B | 14G | Married | $50,000 - $75,000 | Somewhat concerned | | Detached house | | Mother | |  |
| NS3 | 3 | 7 | G | 4B | Married | $50,000 - $75,000 | Somewhat concerned | | Detached house | | Mother | |  |
| NS4 | 3 | 11 | G | 9G | Married | $150,000- $250,000 | Somewhat concerned | | Detached house | | Mother | |  |
| NS5 | 1 | 11 | B | None | Separated | $50,000 -$75,000 | Not concerned | | Low rise Apartment/Condo | | Mother | |  |
| NS6 | 1 | 10 | B | 18G, 20G | Widowed | unknown | Somewhat concerned | | Detached house | | Mother | |  |
| NS7 | 2 | 8 | B | 8B | Married (away) | unknown | Somewhat concerned | | Detached house | | Mother | |  |
| NS8 | 1 | 11 | G | 13B | Married | unknown | Somewhat concerned | | Detached house | | Mother | |  |
| NS9 | 3 | 12 | B | 12B, 2, NB | Married | unknown | Somewhat concerned | | Detached house | | Father | |  |
| NS10 | 2 | 10 | B | 8B | Married | unknown | | Very Concerned | | Detached House | | Father | |
| NS11 | 1 | 8 | G | 5G, 2G | Common law | | | Somewhat concerned | |  | | Mother | |
| NS12 | 3 | 9 | G | None | single, never married | unknown | | Somewhat concerned | | Detached house | | Mother | |
| NS14 | 1 | 10 | B | None | married | unknown | | Very concerned | | low-rise apartment | | Father | |
| NS15 | 1 | 9 | G | None | single, never married | unknown | | Not concerned | | camper home | | Mother | |
| NS16 | 2 | 9 | B | 7G, 3B | married | unknown | | Very concerned | | detached house | | Mother | |

BC: British Columbia; ON: Ontario; NS: Nova Scotia. G: Girl; B: Boy.
